# Correlation between Sperm Parameters and Protein Expression of Antioxidative Defense Enzymes in Seminal Plasma: A Pilot Study

**DOI:** 10.1155/2015/436236

**Published:** 2015-01-27

**Authors:** Biljana Macanovic, Milica Vucetic, Aleksandra Jankovic, Ana Stancic, Biljana Buzadzic, Eliana Garalejic, Aleksandra Korac, Bato Korac, Vesna Otasevic

**Affiliations:** ^1^The Clinic of Gynecology and Obstetrics “Narodni front”, 11000 Belgrade, Serbia; ^2^Department of Physiology, Institute for Biological Research “Sinisa Stankovic”, University of Belgrade, 11000 Belgrade, Serbia; ^3^Institute of Zoology and Center for Electron Microscopy, Faculty of Biology, University of Belgrade, 11000 Belgrade, Serbia

## Abstract

*Background*. Semen analysis is the cornerstone in the evaluation of male (in)fertility. However, there are men with normal semen tests but with impaired fertilizing ability, as well as fertile men with poor sperm characteristics. Thus, there is rising interest to find novel parameters that will help to predict and define the functional capacity of spermatozoa. *Methods*. We examined whether there is a correlation between semen parameters (count, progressive motility, and morphology) and protein expression/activity of antioxidative defense enzymes in seminal plasma from 10 normospermic subjects. *Results*. Sperm progressive motility was in positive correlation with seminal plasma protein expression of both superoxide dismutase (SOD) isoforms (MnSOD and CuZnSOD) and catalase. Also, positive correlation was observed between sperm count and MnSOD protein expression, as well as between sperm morphology and protein expression of catalase in seminal plasma. In contrast, protein expression of glutathione peroxidase was not in correlation with any sperm parameter, while its activity negatively correlated with sperm morphology and motility. *Conclusions*. These data suggest that evaluation of protein expression of antioxidative defense enzymes in seminal plasma might be of importance in the evaluation of male fertility status and that could be used as an additional biomarker along with classic semen analysis in assessment of semen quality.

## 1. Introduction

Infertility is one of the most serious medical problems worldwide. There has been dramatic increase in male infertility over the last years, and many experts in the field predict that infertility rate could double over the next decade. Nearly 40% of the issues involved with infertility are attributable to a male factor [1]. Modern lifestyle and social and environmental factors account for part of the decrease in male reproductive capability.

Fundamental parameters that ascertain the functional ability of spermatozoa are sperm count, motility, and sperm morphology [2]. Analysis of these parameters is commonly used to determine/predict fertility status of the men. Most men investigated for infertility have lower-than-normal number of sperms in the semen, or adequate number, but reduced sperm motility (asthenozoospermia), abnormal morphology (teratozoospermia), or combination of both. However, almost 30% of the male factor infertility cases have no known cause, indicating the lack of sensitive semen tests for the diagnosis of infertility. In addition, there are men with normal semen tests (normozoospermia) but with impaired fertilizing ability of the sperm and inability to achieve pregnancy. On the other hand, there are men with poor sperm characteristics who have no problem to achieve fertilization. This clearly points out that evaluation of fertilizing potential based only on semen parameters is not sufficient to determine fertility status of the men. From the above it arises also that etiology of infertility is poorly understood and that events on molecular level in both spermatozoa and seminal plasma could be important for male infertility.

For these reasons, one of the most important aims in reproductive biology today is determination of new potential biomarkers of male infertility. It has been shown recently that seminal plasma proteins could serve as important biomarkers for male infertility [3]. In addition, functional proteomic analysis revealed proteins that are over- or underexpressed in the seminal plasma of men with poor semen quality.

Among proteins necessary for sperm function and survival, seminal plasma is endowed with numerous enzymatic antioxidants such as superoxide dismutase (SOD), catalase and glutathione peroxidase (GSH-Px), and a variety of nonenzymatic antioxidants such as ascorbate, urate, *α*-tocopherol, pyruvate, and less amounts of glutathione. This protection compensates for the loss of cytoplasmic sperm enzymes that occurs during maturation and transportation processes, resulting in diminution of the spermatozoa's endogenous enzymatic and repair defenses [4–7].

Different studies have investigated relationship between the seminal plasma antioxidative enzymes and sperm motility as well as specific ejaculate pathologies, that is, quality, but the results are controversial [4, 8–15] and, up to now, such lack of consensus keeps the debate open.

In line with this, in this pilot study we try to gain insight on the mechanisms of male infertility from the perspective of the possible importance of protein expression profile of antioxidative defense (AD) enzymes in seminal plasma and spermatozoa functional parameters.

## 2. Materials and Methods

### 2.1. Samples

The Ethic Committee of the Clinic for Gynecology and Obstetrics “Narodni front” and of the Institute for Biological Research at the University of Belgrade approved the study. Semen samples from ten subjects, who signed an informed consent, were classified as normospermic, according to criteria established by the WHO [2]. The mean of participants' age and semen parameters are summarized in Table 1. These subjects were recruited from couples applying for reproductive technology procedures at the Artificial Reproductive Technology Department of the Clinic for Gynecology and Obstetrics “Narodni front”, Belgrade, Republic of Serbia, with an infertility caused by woman's factor. The seminal samples were obtained by masturbation after 3–5 days of abstinence. After complete liquefaction (average time 20 min), manual semen analysis was performed and clear seminal plasma was separated from the sperm pellet by centrifugation at 3000 g for 30 minutes to ensure complete removal of the cellular components. The supernatants obtained were aliquoted and stored at −80°C until subsequent analysis.

### 2.2. SDS-PAGE and Western Blotting

Western blots were conducted as described previously [16], using antibodies against manganese SOD (MnSOD) (ab13533; 1 : 5000), copper, zinc SOD (CuZnSOD) (ab13498; 0.2 g l−1), catalase (ab1877; 1 : 1000), and GSH-Px (ab16798; 1 : 2000) (all purchased from Abcam, Cambridge, UK). Quantitative analysis of immunoreactive bands was conducted with ImageQuant software. Volume was the sum of all the pixel intensities within a band, that is, 1 pixel = 0.007744 mm^2^. We averaged the ratio of dots per band for the target protein in corresponding samples, from three similar independent experiments. Data were then statistically analyzed.

### 2.3. Activity of AD Enzymes

SOD activity was determined by the method of Misra and Fridovich [17] but at 26°C and expressed in U mg^−1^ protein. Total specific SOD activity and MnSOD activity after the inhibition with 4 mM KCN were measured and then CuZnSOD activity was calculated. SOD units were defined as the amount of the enzyme inhibiting epinephrine oxidation by 50% under the appropriate reaction conditions. Catalase was assayed according to Beutler [18] and the activity expressed in *μ*mol H_2_O_2 _min^−1 ^mg^−1^ protein. GSH-Px was determined using* t*-butylhydroperoxide as a substrate [19] and the activity expressed in nmol NADPH min^−1 ^mg^−1^ protein. GST was measured by the method of Habig et al. [20] and the activity is expressed in nM GSH min^−1 ^mg^−1^ protein.

### 2.4. Additional Assays and Statistical Analysis

Protein content was estimated using bovine serum albumin as reference protein [21]. Pearson correlation coefficient was used to evaluate the relationship of AD enzymes protein expression/activity with different sperm parameters. All data are presented as mean ± S.E.M. *P* values less than 0.05 were considered statistically significant.

## 3. Results

Table 2 shows positive correlation (*r* = 0.843, *P* < 0.01) between spermatozoa count per mL and protein expression of MnSOD in seminal plasma of corresponding samples. Also, a strong positive correlation between sperm progressive motility and protein expression of CuZnSOD (*r* = 0.908, *P* < 0.01), MnSOD (*r* = 0.797, *P* < 0.01), and catalase (*r* = 0.789, *P* < 0.01) as well as between sperm morphology and protein expression of catalase in seminal plasma of corresponding samples (*r* = 0.603, *P* < 0.1) is shown in Table 2.

Table 3 summarizes relationship between sperm parameters and activity of antioxidant enzymes in seminal plasma of corresponding samples. Highly negative relationship was observed between seminal plasma GSH-Px activity and sperm progressive motility (*r* = −0.705, *P* < 0.05), that is, morphology (*r* = −0.601, *P* < 0.1).

## 4. Discussion

In the present pilot study we have shown that protein expression of CuZnSOD, MnSOD, and catalase in seminal plasma is in strong positive correlation with some of semen quality parameters. Also, negative relationship between seminal plasma GSH-Px activity and sperm morphology and progressive motility has been shown. These data shed a new light on odd players, seminal plasma antioxidative components, as potential molecular markers of male fertility status.

Relationship between the seminal plasma antioxidative enzymes activity and sperm quality has been investigated so far, but the results obtained are controversial [4, 8–15]. Our finding of negative correlation between seminal plasma GSH-Px activity and sperm morphology is supported by the data of Atig et al. [22]. On the other hand, negative correlation between seminal plasma GSH-Px activity and sperm motility detected in our study is in discordance with the data reported by the same authors [22]. Opposite relationship, that is, positive correlation between seminal plasma GSH-Px activity and sperm quality, was also found by Giannattasio et al. [23], Dandekar et al. [24], and Hsieh et al. [25]. Such variability and controversy of the findings, along with absence of any correlation between sperm parameters and SOD and catalase activity in seminal plasma which is shown here, strongly indicate that the activity of AD enzymes in seminal plasma is not a useful tool in predicting fertilizing status of men, at least in the normospermic samples.

On the other hand, here we have shown very good correlation between protein expressions of AD enzymes in seminal plasma with semen parameters. Thus, these data suggest that protein expressions of AD enzymes in seminal plasma could be used as predictor of male fertility status. Namely, we demonstrated positive correlation between protein expression of (a) MnSOD and sperm count and progressive motility, (b) catalase and sperm motility and morphology, and (c) CuZnSOD and sperm morphology. This strong correlation of MnSOD, CuZnSOD, and catalase expression and sperm quality parameters clearly illustrates importance of these proteins (at least some of them) in male (in)fertility. In the light of recently shown importance of seminal plasma proteins in sperm functioning, these data might be very important and certainly warrant the attention, in terms of molecular markers of male infertility, in particular.

This is noteworthy from the standpoint of finding new approach in determining semen quality apart of semen analysis, that is, increasing number of factors that can provide information about semen quality, especially in the case of idiopathic male infertility. In addition, this also could be helpful for selection of the most potent spermatozoa for* in vitro* fertilization and subsequent improvement of assisted fertilization outcome. One more important fact is that protein expression of AD enzymes in seminal plasma is determined by Western blot that is noninvasive, simple, and easy applicable method in most labs.

In general, presented data suggest that evaluation of protein expression of AD enzymes in seminal plasma might be of prognostic importance in the evaluation of male fertility status and that could be used as an additional biomarker along with classic semen analysis in assessment of semen quality. We believe that these data will help the development of new approaches to diagnose and treat male infertility. Certainly, further study using larger sample size, spermatozoa with different pathologies, and semen samples from infertile men is needed to validate these findings.

## Figures and Tables

**Table 1:** The mean participant's age and semen parameters.

Parameters	Average ± S.E.M. (of 10 samples)	Min/max	Lower reference limit
Age of donors (y)	31.80 ± 1.82	25/34	NA
Abstinence time (day)	4.10 ± 0.44	3/5	2
Volume of semen (mL)	3.37 ± 0.19	2.5/4.5	1.5
Number of spermatozoa (∗10^6^/mL)	81.90 ± 8.84	30/130	>15
Motility (%)	60.00 ± 2.01	50/70	>40
Normal morphology (%)	36.60 ± 2.09	30/50	>4
Number of leukocytes	3.10 ± 0.69	2/6	<6
Number of erythrocytes	(—) absent	(—) absent	(—) absent

**Table 2:** Correlation between sperm parameters and relative protein expression of antioxidative enzymes in seminal plasma.

	CuZnSOD	MnSOD	GSH-Px	Catalase
Number of spermatozoa (×10^6^)	*r* = 0.538 *P* = 0.109 NS	*r* = 0.843 *P* = 0.002 ∗∗∗	*r* = 0.359 *P* = 0.308 NS	*r* = 0.502 *P* = 0.139 NS

Progressive motility of spermatozoa (%)	*r* = 0.908 *P* = 0.0003 ∗∗∗	*r* = 0.797 *P* = 0.006 ∗∗∗	*r* = 0.403 *P* = 0.248 NS	*r* = 0.789 *P* = 0.007 ∗∗∗

Morphology of spermatozoa (%)	*r* = 0.434 *P* = 0.210 NS	*r* = 0.307 *P* = 0.388 NS	*r* = −0.118 *P* = 0.248 NS	*r* = 0.603 *P* = 0.066 ∗

CuZn/Mn SOD, CuZn/Mn superoxide dismutase; GSH-Px, glutathione peroxidase.

Significance: NS, not significant; ^*^ *P* < 0.1; ^***^ *P* < 0.01.

**Table 3:** Correlation between sperm parameters and activity of antioxidative enzymes in seminal plasma.

	CuZnSOD	GSH-Px	GST
Number of spermatozoa (×10^6^)	*r* = 0.094 *P* = 0.796 NS	*r* = −0.179 *P* = 0.621 NS	*r* = 0.422 *P* = 224 NS

Progressive motility of spermatozoa (%)	*r* = 0.288 *P* = 0.420 NS	*r* = −0.705 *P* = 0.023 ∗∗	*r* = 0.255 *P* = 0.477 NS

Morphology of spermatozoa (%)	*r* = 0.405 *P* = 0.246 NS	*r* = −0.601 *P* = 0.066 ∗	*r* = 0.314 *P* = 0.377 NS

CuZnSOD, CuZn superoxide dismutase; GSH-Px, glutathione peroxidase; GST, glutathione S-transferase.

Significance: NS, not significant; ^*^ *P* < 0.1; ^**^ *P* < 0.05.
